# What’s so special about special issues: Highlighting a central role of *parasitology* to support specific innovations and advance progress within our discipline

**DOI:** 10.1017/S0031182025000125

**Published:** 2025-01

**Authors:** John Ellis, Cinzia Cantacessi, J. Russell Stothard

**Affiliations:** 1 School of Life Sciences, University of Technology Sydney, Ultimo, NSW 2007, Australia; 2 Department of Veterinary Medicine, University of Cambridge, Madingley Road, Cambridge, CB3 0ES, UK; 3 Department of Tropical Disease Biology, Liverpool School of Tropical Medicine, Liverpool L3 5QA, UK

**Keywords:** academic publishing, editorial integrity, guest editors, open access, special issues

## Abstract

**Figure:**
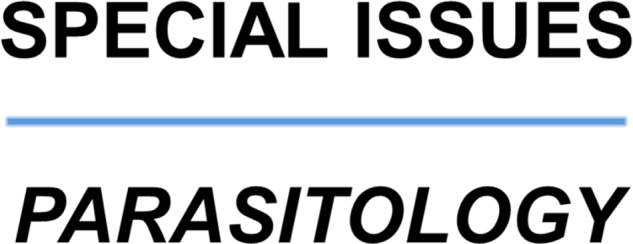

## Introduction

Special issues in *Parasitology* have always proven popular with our readers. Historically, these have covered a diverse set of topics, addressing either specific or broad themes, within our chosen discipline. Looking ahead and taking a broader point of view in this editorial, we consider their academic value and future significance. This is especially important today, and now more than ever, because of their considerable importance they bring to scholarly debate. In a wider context of scientific publishing, however, recent publications have raised debate, both with positive and negative features, of the role that special issues can play (Gleasner and Sood, 2024). Here, we add to the debate and highlight some of the more important issues particularly pertinent to *Parasitology* below. Foremost, the provocative review by Gleasner and Sood (Gleasner and Sood, 2024) suggested that reputable academic journals should be more transparent about their editorial processes behind the commissioning or hosting of special issues. As such, we highlight them here, especially for the benefit of our loyal readers and community members, to better illustrate our intentions.

## The value of a special issue: Are all viewpoints considered?

Special issues (which can also go by other names such as ‘themed issues’ or ‘supplements’ in other journals) typically focus on select discipline areas (Olk and Griffith, 2004). In so doing, they can raise the global profile of a journal through attracting some of the best authors (and reviewers), which thereby increases their articles’ and hosting journal’s citations (Conlon et al., 2006; Sainte-Marie et al., 2020). Rises in such bibliometrics, for example in downloads or impact factors (Repiso et al., 2021), is tangible evidence of better outreach and academic impact. Indeed, having a collection of contemporary papers addressing a common theme also helps to spark the flames of academic interest and further study, even if its authors were working in international competition rather than collaboration.

Despite desirable benefits of special issues, previously there have been concerns that special issues could be seen as undermining research integrity (Mills et al., 2024; Wright, 2024). Whilst financial drivers to keep a journal buoyant are always influential considerations in journal management, we are pleased to say that for *Parasitology*, we have navigated the financial transition to open access wisely. We have always strived a better balance to service the needs of authors, readers and publisher, ensuring that current open access agreements and publishing responsibilities are pragmatic, useable and far reaching (Ellis and Stothard, 2024).

## The how and why of special issues in *parasitology*

**Figure 1.**
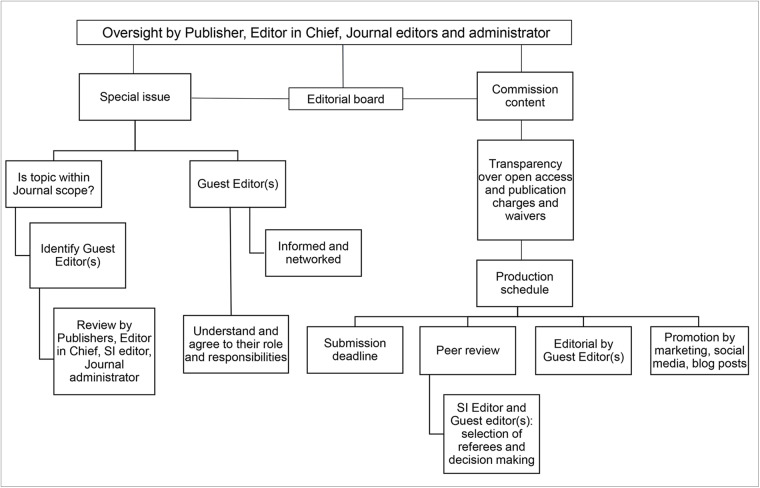
Flow diagram showing the broad considerations behind publishing a special issue in Parasitology.

The Council on Publication Ethics (COPE) have provided helpful guidelines on best practices for the production of special issues that are of significant value to the scientific and broader community (Anonymous, 2023). At *Parasitology*, we endeavour to maintain integrity associated with special issue articles (indeed all articles) using a variety of quality control procedures, that follow closely COPE’s guidelines (Figure 1). Our ambition moving forward, as it has been before, is to allocate two issues each year, from the 14 produced annually, for specialist topics and themes.

Oversight of special issues is made by the Publishing Editor of Cambridge University Press & Assessment (Alison Paskins), along with the Journal’s Editor-in-Chief (Prof Russell Stothard) and the Special Issues Editor (Em/Prof John Ellis). The inaugural editor responsible for special issues was Prof Les Chappell who assumed the role in 2011, having spent many years as a co-ordinating editor (from 1990). Prof Ellis joined the team in 2015 as Special Issues Editor following Les’ ill-health. Subsequent appointment of Guest Editors to a special issue by this management team is not taken lightly, as the project relies heavily on a Guest Editors academic standing and their essential network of professional contacts. It also allows training opportunities for those Guest Editors (whom may be inexperienced as an editor) to develop editorial skills under supportive supervision of the editorial team. These synergistic working relationships have had many mutual benefits.

Our first consideration for selection of a special issue is that it must fit the foundation scope of *Parasitology*, aligning with medical, veterinary or wildlife themes (https://www.cambridge.org/core/journals/parasitology/information/about-this-journal). Second, the featured topic and theme should be of significant scientific interest, or provide novel education value, to our readership. Third, which is a more intangible element, involves the academic kudos and mentorship role for its guest editor(s), contributing to solidify a network of submitting authors, some of whom may be early career researchers themselves or PhD students. In selection of the theme, several strategies are used to identify topics for special issues in the face of fierce competition provided by other scientific journals. *Parasitology* historically, since 1989, has published a Special Issue resulting from the Autumn Symposium of the British Society for Parasitology (BSP). The first issue produced in collaboration with the BSP was published as a supplement on ‘*Vaccines and vaccination strategies*’ (Volume 98, Issue S1). Another volume on ‘*Research developments in the study of parasitic infections*’ was specifically identified as a special issue (Anderson et al., 1989). Since then, 68 special issues have appeared in *Parasitology*. A forthcoming special issue on ‘*Ticks and tick-borne diseases*’ will be number 69.

**Figure 2.**
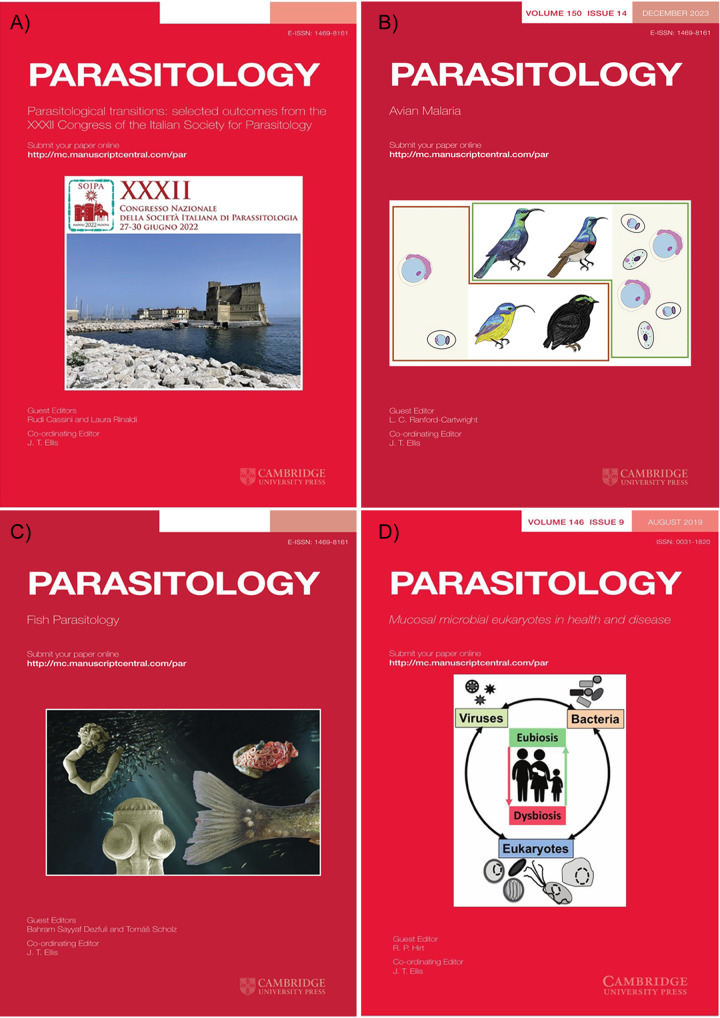
Front covers of four special issues: (A) XXXII congress of the Italian society for parasitology; (B) avian malaria; (C) fish parasitology; (D) mucosal microbial eukaryotes in health and disease.

In addition to our collaborative efforts with the BSP whereby a physical meeting of the society was supported, *Parasitology* uses a variety of procedures to identify topics for special issues. Our journal has been partnering with additional academic societies aligned with our objectives to produce special issues of more international value and global outreach. Recent examples include the meetings organised by the Italian (Figure 2A) and Malaysian Society’s. Alternatively, to engage with our authorship and readership, open call for papers on specialist topics of interest was also introduced in 2022. This strategy has been successful with two issues on ‘*Avian malaria*’ (Guest Editor Prof Lisa Ranford-Cartwright) (Figure 2B) and ‘*Ticks and tick-borne diseases*’ (Guest Editor Prof Ala Tabor).

*Parasitology* often seeks suggestions for special issues from its editorial board and a recent example is ‘*Fish Parasitology*’ from Guest Editors Profs Bahram Sayyaf Dezfuli and Tomáš Scholz (Figure 2C). To engage more widely, suggestions from readers of *Parasitology* are always welcome, and an up-coming example will be the call for papers on ‘*Parasites of the Brain*’ (Guest editors Profs Stefania Zanet and Carlo Contini). We engage with the outcomes of international conferences when the opportunities arise (Figure 2D) as they are known to provide additional academic impacts. Two current activity’s arose from the International Congress for Tropical Medicine and Malaria (ICTMM2024) organised by the Malaysian Society for Parasitology and Tropical Medicine; two open calls will emerge in early 2025 the first ‘*Parasites of the genital tract: Short- and long-term consequences’* is already online (https://www.cambridge.org/core/journals/parasitology/announcements/call-for-papers/parasites-of-the-genital-tract-and-their-short-and-long-term-consequences) with the second ‘*Primate malaria and zoonosis*’ soon to appear. The Guest Editors of the former are Prof Janelisa Musaya and Dr Seke Kayuni while the latter is Prof Lucas Van Lun. We have also introduced a short video snippet from the editors to help frame the ambition of the special issue as another way to engage and encourage authors and their teams.

## Capturing and managing future interests in special issues

Consideration of bibliometric studies in the discipline of parasitology provides valuable supporting evidence for choosing topics for a special issue. Such studies help define the core and emerging areas of parasitology, which in themselves will be overlapping. Investigative tools such as SciMAT, VOSviewer and SWIFT-Review are particularly useful in providing a detailed overview of scholarly activity (Ellis et al., 2020). Furthermore, the use of evolution (longitudinal) maps (Cobo et al., 2012) in such tools represent an underutilised approach to help define and frame emerging areas for consideration within a special issue. Similarly, cursory surveys of certain social media platforms, though continuously changing, can also be helpful in this context, largely due to their relative immediacy (Ellis and Reichel, 2023).

Generative AI methods are emerging at a frightening rate and their use in all areas of scientific research, from conception to publishing, are gaining traction (Susarla et al., 2023; Pu et al., 2024). Cambridge University Press & Assessment provides policies for guidance around the use of AI in research papers published in all of its journals (https://www.cambridge.org/core/services/publishing-ethics/authorship-and-contributorship-journals). Despite being a potentially disruptive technology and a threat to research integrity, it is essential that the use of GenAI be used wisely in all areas of *Parasitology* and given the proper consideration it deserves. Whilst machine learning algorithms digest and assimilate scientific content, but never as nuanced as actual users can, we remember that much of *Parasitology*’s function is to inform and educate the readership. Our ambition here is to help to create supportive networks of academics and related stakeholders that share new common ground.

One of the most important considerations of a special issue is a friendly negotiation with Guest Editors loosely along the expected timeline for eventual production of a special issue. This considers all parts of the schedule from receiving submissions to the final publication of the special issue. We consider and must anticipate the risks associated with the production of a special issue such as ‘drop outs’ (authors who agree to provide a paper but then do not once they realise the demands on their *pro bono* time), late submission of papers beyond an agreed submission date, various staff illnesses, time of year and so on. Similarly, the difficulty in finding suitable referees for submitted papers is increasing, a common issue across scientific publishing in general. Could future AI algorithms be repurposed to fulfil this future function, we sincerely hope not, though there are useful automatons that detect overt plagiarism.

In *Parasitology*, we take a formal step each year to thank our referees and in 2024 over 300 referees contributed their time and effort to the review of papers in Parasitology (see the Supplementary Material for the full list). We thank them for their incredibly valuable contributions to the journal’s peer-review processes. Indeed, peer review of special issue papers are an additional expectation to be evaluated as fairly and transparently by those with appropriate experience and credentials to do so. It follows, in part, the identical process for our regular articles but accommodates more specialist requirements. However, special issue articles receive no ‘*special*’ treatment as all articles have to be sufficient in clarity and quality for publication. All peer-review processes are carried out in ScholarOne (https://mc.manuscriptcentral.com/par). Consequently, article turnaround times in *Parasitology* are very similar for special issue versus regular issues. Guest Editors are intimately involved in the special issue decision making from the very beginning by inviting authors to contribute papers, to selection of referees and manuscript decision making. Guest Editors are also expected to contribute an editorial preface and social media blog for the special issue in a timely manner. This politely recognises the wider contributions to knowledge of these manuscripts. We acknowledge here the great value our Guest editors bring to the special issue, and we are of course very grateful to them for their time, expertise and commitment.

## A new era of open access and institutional interactions

The transition of *Parasitology* to a Gold Open Access model has been previously described in these pages (Stothard and Ellis, 2022) and details are readily available on the publishers web site (https://www.cambridge.org/core/eligibility-checker). Since the readership base of *Parasitology* is truly worldwide, it is important to ensure that no author is excluded from publishing in *Parasitology* because of the cost of article publishing charges. Inclusivity is an essential consideration of *Parasitology* publishing and this also applies to special issues, where discretionary waivers can be granted upon reasonable request. For authors in low- and middle-income countries who do not have access to funding to pay APCs, Cambridge University Press & Assessment have several options available to support authors (https://www.cambridge.org/core/open-research/funding-open-access-publication). Through a special issue targeted at early career researchers in South America and led by Dr Carolina Verrisimo (University of Galway) as the Guest Editor, we endeavour to highlight this important need for inclusiveness across the world and its many nations.

This year past, *Parasitology* also explored a new avenue to explore the commissioning of a special issue. This springboarded from the close relationship with Cambridge University Press & Assessment with the University of Cambridge itself. Under the leadership of Senior Editor Prof Cinzia Cantacessi, *Parasitology* recently joined forces with the Infection & Immunity Theme of the University of Cambridge’s School of Biological Sciences to host a ground breaking symposium, ‘*Miniature Worlds: Organoid Research in Parasitology*’ (https://www.cambridge.org/core/journals/parasitology/announcements/events/miniature-worlds-organoid-research-in-parasitology). This hybrid event, held on November 8th, 2024 at the West Cambridge Hub, drew over 150 leading experts and early-career researchers. It delved into the exciting world of cultivating parasite organoids – miniature, self-organizing structures that mimic complex organs – and the unprecedented opportunities they present.

Four esteemed speakers spearheaded the symposium: Dr Maria Duque-Correa (Cambridge Stem Cell Institute, Cambridge) provided an overview of the application of gut organoids to understand the complex interactions between the intestinal epithelium and whipworms; Dr David Smith (Moredun Research Institute, Edinburgh) presented recent progress on the establishment of ovine intestinal organoids for molecular studies of key nematodes infecting livestock species; Dr Mattie Pawlowic (University of Dundee, Scotland) focused on the development of intestinal organoids for studies of the fundamental biology of *Cryptosporidium*; finally, Prof John Dalton (Galway University, Ireland), illustrated the immense potential of utilising hepatic spheroids to study host–parasite interactions over the course of early infection by liver flukes.

Further presentations explored novel data on utilizing intestinal organoids to elucidate immune-molecular mechanisms in host–parasite interactions. These included studies on gastrointestinal nematode infections (Prof Collette Britton and Dr Matias Gaston-Perez, University of Glasgow), tick infestations (Dr Jan Perner, Czech Academy of Sciences), and *Leishmania* infection models (Dr Rens Zonneveld, Amsterdam University Medical Center).

## An outlook on the speciality of special issues

As the field enters a new era, *Parasitology* invites researchers to submit their work on organoid-based studies to our forthcoming special issue dedicated to this exciting frontier. By fostering knowledge sharing and collaboration, we can accelerate the development of innovative solutions to combat the global health challenges posed by parasitic infections. We are hopeful our pipeline model of hosting an event, every other year in Cambridge, will become an important date in the parasitology calendar.

Given the emergence of a huge increase in the number of special issues commissioned in the discipline of parasitology, it is essential, going forward, that this journal considers the question on how topics and Guest Editors are selected for special issues. On this point, Huang and colleagues recommended ‘*optimising the procedures for selecting special issue topics and reviewing submissions*’ (Huang et al., 2022), mainly because they observed the presence of many sub-standard papers in the special issues they examined. Clearly, we will avoid publishing sub-standard papers at all costs, however optimising procedures for selection of special issue topics is clearly worthwhile. On this point, it is likely that we will look to the vast experience and diversity of the journal’s editorial board and readership for assistance in this endeavour.

To close, it is essential to always ensure the research integrity and trust in the papers published in *Parasitology*. We certainly endorse and agree with the general recommendations (Gleasner and Sood, 2024) and guidance from COPE and the International Committee of Medical Journal Editors will be used to ensure the quality and integrity of all articles published. Our peer-review processes are and will remain certainly stringent. Nevertheless, there is always room for improvement and Cambridge University Press & Assessment along with its journal *Parasitology* will hope to lead by example in this discipline.

## Supporting information

Ellis et al. supplementary material Ellis et al. supplementary material

## Editors

Russell Stothard, *Editor-in-Chief*, 2020 –

John Ellis, *Deputy Editor-in-Chief & Special Issues Editor*, 2021 –

Cinzia Cantacessi, 2022 –

Joseph Jackson, 2022 –

Laura Rinaldi, 2020 –

Lisa Ranford-Cartwright, 2021 –

Jonathan Wastling, 2011 –

## Social media editors

Emily Pascoe, *Senior Social Media Editor*, 2022–2025

Joel Barratt, 2023–2024

Derrick Osakunor, 2022–2024

Timothy J Odom, 2024–

Angus O’Ferrall, 2024–

## Supplementary material

The supplementary material for this article can be found at https://doi.org/10.1017/S0031182025000125.
