# Supplementary material for: What’s so special about special issues: Highlighting a central role of parasitology to support specific innovations and advance progress within our discipline
Source: Parasitology. 2025 Apr 14;152(1):1–5. doi: 10.1017/S0031182025000125 (PMC12088914; doi:10.1017/S0031182025000125)
Supplement: Ellis et al. supplementary material [file S0031182025000125sup001.docx]

**List of Reviewers, 2024**

*Parasitology* would like to thank the following people, who kindly provided reviews for the journal in 2024:

Hassan Abdi Hussein, Jigjiga University, Ethiopia

Tyler Achatz, Middle Georgia State University, United States

Edson Adriano, Universidade Federal de São Paulo, Brazil

Haroon Ahmed, COMSATS Institute of Information Technology, Pakistan

Kavita Ahuja, Guru Jambheshwar University of Science and Technology, India

Nahid Ali, Indian Institute of Chemical Biology, India

Nazmiye Altintas, Ege University, Turkey

Gertraut Altreuther, Elanco Animal Health GmbH, Germany

Cristian A. Alvarez Rojas, Pontificia Universidad Catolica de Chile, Chile

Linda Amoah, University of Ghana, Ghana

Renato Andreotti, Embarapa, Brazil

Daniela de Angeli Dutra, University of Otago, Brazil

John Archer, Natural History Museum, UK

Rossana Arroyo, CINVESTAV-IPN, Mexico

Spiridoula Athanasiadou, SRUC Edinburgh Campus, UK

Stephen Atkinson, Oregon State University, United States

Mehmet Aykur, Ege University, Turkey

Carlos Azevedo, University of Porto, Portugal

Francisco Javier Aznar, University of Valencia, Spain

Nlingisisi Babayani, University of Botswana, Botswana

Stephen Barker, The University of Queensland, Australia

Joel Barratt, Centers for Disease Control and Prevention, United States

Luiz Daniel de Barros, Federal University of Lavras, Brazil

Diane Barton, Charles Sturt University - Wagga Wagga Campus, Australia

Kelly Bateman, Cefas, UK

Daniel Becker, Montana State University System, United States

Jerzy Behnke, University of Nottingham, UK

Salem Belkessa, Ziane Achour University of Djelfa, Algeria

Jeffrey Bell, University of North Dakota, United States

Michal Benovics, Masaryk University, Czech Republic

Ian Beveridge, University of Melbourne Faculty of Veterinary and Agricultural Sciences, Australia

Chet Bhatta, Radford University, United States

Felipe Bisaggio Pereira, Universidade Federal de Mato Grosso do Sul, Brazil

David Blair, James Cook University, Australia

Jaime Blair, Franklin and Marshall College, United States

Isabel Blasco-Costa, Museum d'histoire naturelle de la Ville de Geneve, Switzerland

Jamie Bojko, Teesside University, UK

Nathan Bott, RMIT University School of Science Cluster, Australia

Richard Bradbury, James Cook University, Australia

Rod Bray, Natural History Museum, UK

Joshua Brian, King's College London, UK

Charles Addoquaye Brown, University of Ghana College of Health Sciences, Ghana

Francesco Buono, University of Naples Federico II, Italy

David Bushek, Rutgers University, United States

Javier A. Bustos, Universidad Peruana de Ciencias Aplicadas (UPC), Peru

Alejandro Cabezas-Cruz, Ecole Nationale Veterinaire d'Alfort (ENVA), France

Rafael Calero-Bernal, Complutense University of Madrid, Spain

Delfina Cantatore, Instituto de Investigaciones Marinas y Costeras, Facultad de Ciencias Exactas y Naturales. Universidad Nacional de Mar del Plata-CONICET, Argentina

Chris (Katharine C.) Carter, Strathclyde University, UK

Loren Cassin-Sackett, University of Louisiana at Lafayette, United States

Rudi Cassini, Department of Animal Medicine, Production and Health, Italy

Elisa Castaldo, Federico II University Hospital, Italy

Serena Cavallero, Università degli Studi di Roma La Sapienza, Italy

Sławomir Cerbin, Uniwersytet im Adama Mickiewicza w Poznaniu, Poland

Sulagna Chakraborty, University of Illinois Urbana-Champaign, United States

Abigail Chan, Mahidol University Faculty of Tropical Medicine, Thailand

Lidia Chitimia-Dobler, Bundeswehr Institute of Microbiology, Germany

Anindo Choudhury, St. Norbert College, United States

Arif Ciloglu, Erciyes Universitesi, Turkey

Lavinia Ciuca, University of Naples Federico II, Italy

Christine Clayton, Heidelberg University, Germany

Eduardo Coelho, Universidade Federal de Minas Gerais, Brazil

Valery Combes, University of Technology Sydney, Australia

Bruce Conn, Berry College School of Mathematical and Natural Sciences, United States

Alba Cortes, Facultad de Farmacia-Universidad de Valencia, Spain

Robert Cowie, University of Hawaii, United States

Clay Cressler, University of Nebraska-Lincoln, United States

Thomas Cribb, University of Queensland, Australia

Armando Cruz-Laufer, Hasselt University, Belgium

John Dalton, National University of Ireland Galway, Ireland

Sid Das, University of Texas at El Paso, United States

Georgiana Deak, Universitatea de Stiinte Agricole si Medicina Veterinara din Cluj-Napoca, Romania

Julia Diaz, CONICET, Argentina

Olgica Djurkovic-Djakovic, Institute for Medical Research, University of Belgrade, Serbia

Cecilia Dominguez, IADIZA, Argentina

Sheila Donnelly, University of Galway, Ireland

Quinton Dos Santos, University of Johannesburg, South Africa

Jitender Dubey, USDA, United States

Melanie Duc, Nature Research Centre, Lithuania

Meghan Duffy, University of Michigan, United States

Jenny Dunn, Keele University, UK

William Diymba Dzemo, Walter Sisulu University - Mthatha Campus, South Africa

Marcin Dziuba, University of Michigan, United States

Ananias Escalante, Arizona State University, United States

Anna Faltýnková, Biology Centre Czech Academy of Sciences Institute of Parasitology, Czech Republic

Majid Fasihi Harandi, Kerman University of Medical Sciences, The Islamic Republic of Iran

Francisco Ferreira, Texas A&M University, United States

Peter Fields, University of Basel, Switzerland

Constance Finney, University of Calgary, Canada

Agnes Fleury, Instituto de Investigaciones Biomédicas, Universidad Nacional Autónoma de Mexico, Mexico

Michelle Cristie Fonseca, Instituto Oswaldo Cruz - Fiocruz, Brazil

Sarah Gabriel, University of Ghent, Belgium

Holly Gaff, Old Dominion University, United States

Spencer Galen, University of Scranton, United States

Luz Garcia-Longoria Batanete, University of Extremadura, Spain

Juan Garcia-R, Massey University, New Zealand

Jorge Garrido-Bautista, University of Granada Faculty of Sciences, Spain

Charles Gauci, University of Melbourne, Australia

Annunziata Giangaspero, Università di Foggia, Italy

José Pedro Gil, Karolinska Institute, Sweden

Selma Giorgio, Biology Institute - University of Campinas UNICAMP, Brazil

Patrick Giraudoux, University of Franche-Comté, France

Michał Glądalski, University of Lodz, Poland

Geoffrey Gobert, Queen's University Belfast, UK

Alex Gofton, CSIRO, Australia

Luis Gomez-Puerta, Universidad Nacional Mayor de San Marcos, Peru

Marcelo González-García, Universidad Nacional Autónoma de México, Mexico

Catherine Gordon, QIMR Berghofer Medical Research Institute, Australia

Carlos Graeff Teixeira, Pontifia Universidade Catolica do Rio Grande do Sul, Brazil

Omar Hamarsheh, Al-Quds University, State of Palestine

Patrick Hamilton, University of Exeter, UK

William Harnett, University of Strathclyde, UK

Adam Hayward, Moredun Research Institute, UK

Alexis Heckley, McGill University, Canada

Petr Heneberg, Charles University, Czech Republic

Oscar Hernandes Cordoba, INECOL, Mexico

Zully Hernández Russo, University of the Republic Uruguay, Uruguay

Geoff Hide, Salford University, UK

Barbara Hinney, University of Veterinary Medicine Vienna, Austria

Robert Hirt, Newcastle University, UK

Jane Hodgkinson, Liverpool School of Tropical Medicine, UK

Keith Hopper, USDA-ARS, United States

William Horsnell, University of Exeter Faculty of Health and Life Sciences, UK

Herve Hoste, INRAE, France

Wei Huang, Fujian Agriculture and Forestry University, China

Xi Huang, Beijing Normal University, China

Abdul Jabbar, University of Melbourne, Australia

Susan Jarvi, University of Hawai'i at Hilo, United States

Francisco Jimenez-Ruiz, Southern Illinois University Carbondale, United States

Anja Joachim, University of Veterinary Medicine Vienna, Austria

Nicholas Jonsson, University of Glasgow, UK

Alexandra Juhasz, Liverpool School of Tropical Medicine, UK

Kerstin Junker, Agricultural Research Council, South Africa

Elías Kabbas-Piñango, University of Glasgow, UK

Panagiotis Karanis, University of Cologne, United States

Egil Karlsbakk, University of Bergen, Norway

Nadira Karunaweera, University of Colombo Faculty of Medicine, Sri Lanka

Maria Kazimirova, Institute of Zoology, Slovak Academy of Sciences, Slovakia

Jennifer Keiser, Swiss Tropical Institute, Switzerland

Jane Kelley, Australia Department of Primary Industries and Energy, Australia

Vitaliy A. Kharchenko, II Schmalhausen Institute of Zoology NAS of Ukraine, Ukraine

Safari Kinung'hi, National Institute for Medical Research Mwanza Research Centre, United Republic of Tanzania

Petr Kopacek, Biology Centre Czech Academy of Sciences, Czech Republic

Boris Krasnov, Jacob Blaustein Institutes for Desert Research, Ben-Gurion University of the Negev, Israel

Tiago Kütter Krolow, Universidade Federal do Tocantins, Brazil

Arda Kuyucu, Hacettepe University, Turkey

Yuriy Kuzmin, I I Schmalhausen Institute of Zoology National Academy of Sciences of Ukraine, Ukraine

Yuriy Kvach, Institute of Marine Biology of the NAS of Ukraine, Ukraine

Marcelo Labruna, Universidade de São Paulo, Brazil

Amy Lambert, Centers for Disease Control and Prevention, United States

Maria Latrofa, University of Bari, Italy

Teivi Laurimäe, University of Tartu, Institute of Ecology and Earth Sciences, Estonia

Kara Layton, University of Toronto, Canada

Lydia R. Leonardo, University of the Philippines Diliman, The Philippines

Marshall Lightowlers, The University of Melbourne, Australia

Tim Littlewood, Natural History Museum, UK

Guo-Hua Liu, Hunan Agricultural University, China

Ying Liu, Wannan Medical College, China

Sam Loker, University of New Mexico, United States

Astler Luana, Universidade Estadual de Maringá, Brazil

Pepijn Luijckx, Trinity College Dublin School of Natural Science, Ireland

Geoffrey Lynn, Texas A&M University, United States

Maria E. López-Arellano, Instituto Nacional de Investigaciones Forestales, Agrícolas y Pecuarias, Mexico

Olfat Mahdy, Cairo University Faculty of Veterinary Medicine, Egypt

Laurence Malandrin, INRA, France

Arnaldo Maldonado, Fundação Oswaldo Cruz, Brazil

Ben Mans, University of South Africa, South Africa

Marianna Marangi, Universita degli Studi di Foggia, Italy

Laura Martín-Torrijos, Real Jardín Botánico CSIC, Spain

Carlos Martínez-Carrasco Pleite, Universidad de Murcia - Campus de Espinardo, Spain

Anabel Martínez-Sánchez, Universidad de Alicante, Spain

Josué Martínez-de la Puente, University of Granada, Spain

Dmitri Maslov, Univ. California - Riverside, United States

Simonetta Mattiucci, “Sapienza” University of Rome, Italy

Karen McCoy, CNRS, France

Suzanne McDermott, Seattle Children's Research Institute, United States

Emily McDermott, University of Arkansas, United States

Paul McVeigh, Queen's University Belfast, UK

Rodrigo Megia, University of Porto Research Centre in Biodiversity and Genetic Resources, Portugal

Jairo Alfonso Mendoza-Roldan, University of Bari, Valenzano, Italy, Italy

Farah Haziqah Meor Termizi, Universiti Sains Malaysia, Malaysia

Santiago Merino, Museo Nacional de Ciencias Naturales, Spain

Guilherme Miranda, Instituto Federal de Educacao Ciencia e Tecnologia do Maranhao, Brazil

Piers Mitchell, University of Cambridge, UK

Ian Montgomery, Queen's University Belfast, UK

Carolina Montoya-Ruíz, Universidad Nacional de Colombia, Colombia

Sunil Mor, South Dakota State University, United States

Rodrigo Morchón, University of Salamanca, Spain

Eric Morgan, Queen's University Belfast, UK

Kate Mounsey, University of Sunshine Coast, Australia

Mohamed Abdallah Mohamed Moustafa, Rutgers University New Brunswick, Centre for Vector Biology, United States

Grace Mulcahy, University College Dublin, Ireland

Beatriz Munguía, Universidad de la Republica Uruguay, Uruguay

Kioko Mwikali, KEMRI-Wellcome Trust Research Programme, Kenya

Elmarie Myburgh, Hull York Medical School, UK

Robert Míč, Masaryk University Faculty of Science, Czech Republic

Gunter Müller, Hebrew University of Jerusalem Hadassah Medical School, Israel

Yukifumi Nawa, Faculty of Medicine, Khon Kaen University, Thailand

Masato Nitta, Japan Fisheries Research and Education Agency Fisheries Technology Institute Nansei, Japan

Matthew Nolan, University of Cambridge, UK

Juliana Notarnicola, IBS, CONICET-UNaM, Argentina

Donald Nyangahu, Seattle Children's Research Institute, United States

Elise O’Connell, National Institutes of Health, Laboratory of Parasitic Diseases, United States

Maria Ogrzewalska, Universidade de São Paulo, Brazil

Beth Okamura, Natural History Museum, UK

Colton Padilla, University of Montana, United States

Vaidas Palinauskas, Nature Research Centre, Lithuania

Kishor Pandey, Institute of Tropical Medicine, Nagasaki University, Japan

Rodolfo Paredes, Universidad Andres Bello, Chile

Eunji Park, The University of British Columbia, Canada

Agustin Estrada Pena, University of Zaragoza, Spain

Tom Pennance, Natural History Museum London, UK

Paola Pepe, University of Naples Federico II, Italy

Raul Perez Caballero, IrsiCaixa Institut de Recerca de la Sida, Spain

Ricardo Perez-de-lafuente, Museum of Natural History, UK

Susan Perkins, American Museum of Natural History, United States

Marie-Jeanne Perrot-Minnot, Université de Bourgogne, France

Martin Pfeffer, Leipzig University, Germany

Ronel Pienaar, Agricultural Research Council Onderstepoort Veterinary Research, South Africa

Rafael Pinheiro, University of Campinas Institute of Biology, Brazil

David Poché, Genesis Laboratories Inc., United States

Cecilia Power, RMIT University School of Science Cluster, Australia

Whitney Preisser, University of Washington, United States

Bronwen Presswell, University of Otago, New Zealand

Matias Preza, University of Bern, Switzerland

Keith Price, Pennsylvania Department of Environmental Protection, United States

Iva Přikrylová, University of Limpopo - Turfloop Campus, South Africa

Rupert Quinnell, Leeds University, UK

Amruta Rajarajan, Leibniz-Institute of Freshwater Ecology and Inland Fisheries in the Forschungsverbund Berlin eV, Germany

Angel Ramos, Universidad Veracruzana, Mexico

Lisa Ranford-Cartwright, University of Glasgow, UK

Jose Reck Jr., Rio Grande do Sul Instituto de Pesquisas Veterinárias Desidério Finamor, Brazil

Michael Reichel, Cornell University, United States

Lisa Reynolds, University of Victoria, Canada

Alexis Ribas, University of Barcelona, Spain

Elise Richardson, Freelancer, United States

Philip Riekenberg, NIOZ, Netherlands

Nallely Rivero Perez, Universidad Autónoma del Estado de Hidalgo, Mexico

Rosângela Rodrigues, Universidade Federal de Goias Unidade Academica Especial de Ciencias da Saude, Brazil

Alicia Rojas, University of Costa Rica | UCR Centro de Investigación en Enfermedades Tropicales, Costa Rica

David Rollinson, The Natural History Museum, UK

Bruce Rosa, Washington University School of Medicine in Saint Louis, United States

Andre V. Rubio, Universidad de Chile, Chile

Sonja Rueckert, University of Duisburg-Essen, Germany

Urmas Saarma, University of Tartu, Institute of Ecology and Earth Sciences, Estonia

Joana Santos, University of Basel, Switzerland

Maria Santos, University of Porto Faculty of Sciences, Portugal

Srimonti Sarkar, Bose Institute, India

Bahador Sarkari, Shiraz University of Medical Sciences, The Islamic Republic of Iran

Stefano Scarcelli, University of Naples Federico II, Italy

Henk Schallig, AMC, Netherlands

Gereon Schares, Friedrich-Loeffler-Institut Bundesforschungsinstitut fur Tiergesundheit, Germany

Leonhard Schnittger, Instituto de Patobiología Veterinaria, Centro de Investigaciones en Ciencias Veterinarias y Agronómicas (CICVyA), INTA-Castelar, Argentina

Tomas Scholz, Institute of Parasitology, AS CR, Czech Republic

Andrea Schreiberová, Library of the University of Veterinary Medicine and Pharmacy in Košice, Slovakia

Jessica Schwelm, University of Duisburg-Essen, Germany

W. Evan Secor, Centers for Disease Control and Prevention, United States

Haytham Senbill, Alexandria University, Egypt

Giovanni Sgroi, Istituto Zooprofilattico Sperimentale del Mezzogiorno, Italy

Mohammad Shah Jalal, University of Montreal, Canada

Shookofeh Shamsi, Charles Sturt University, Faculty of Science, Wagga Wagga, Australia

Surendra Sharma, University of North Carolina at Chapel Hill, United States

Aline Silva, National Institutes of Health, United States

Pedro Giovani da Silva, Universidade de Brasilia, Brazil

Neena Singla, Punjab Agricultural University, India

Abhinav Sinha, National Institute of Malaria Research, India

Pavel Široký, University of Veterinary and Pharmaceutical Sciences, Faculty of Veterinary Hygiene and Ecology, Czech Republic

Jan Slapeta, University of Sydney, Australia

Nico Smit, North-West University, South Africa

Maria Soeiro, Instituto Oswaldo Cruz - Fundação Oswaldo Cruz, Brazil

Jacek Sroka, National Veterinary Institute - National Research Institute, Poland

J. Russell Stothard, Liverpool School of Tropical Medicine, UK

Filip Strbac, University of Belgrade, Serbia

Peter Stuart, METLA, Finland

Xun Suo, China Agricultural University, China

Tamara Szentivanyi, HUN-REN Centre for Ecological Research, Hungary

Eszter Szollosi, Eotvos Lorand Tudomanyegyetem Termeszettudomanyi Kar, Hungary

Ala Tabor, The University of Queensland, Australia

Francesca Tamarozzi, Ospedale Sacro Cuore Don Calabria, Italy

Marcos Tavares-Dias, Embrapa Amapá, Brazil

Yuexun Tian, Texas A&M University College of Agriculture and Life Sciences, United States

Juan Timi, Universidad Nacional de Mar del Plata Facultad de Ciencias Exactas y Naturales, Argentina

Paul Torgerson, Vetsuisse Faculty at the University of Zurich, Switzerland

Donato Traversa, Faculty of Veterinary Medicine, University of Teramo, Italy

Elena Tricarico, University of Florence, Italy

Gérald Umhang, Anses Rabies and Wildlife Laboratory, France

M. Adela Valero, University of Valencia, Faculty of Pharmacy, Spain

Jefferson Vaughan, University of North Dakota, United States

José M. Venzal, Universidad de la Republica Uruguay Facultad de Veterinaria, Uruguay

Jozef Vercruysse, University of Ghent. Laboratory of Parasitology, Faculty of Veterinary Medicine, Belgium

Hannah Vineer, University of Liverpool Faculty of Science, UK

Matthew Walker, Southern Illinois University Carbondale, United States

Richard Wall, University of Bristol, UK

Michael Ke Wang, McMaster University, Canada

Marion Wassermann, University of Hohenheim, Germany

Matthew Wayland, University of Cambridge, UK

Ulrike Weber-Stadlbauer, University of Zurich, Switzerland

Nicholas Wee, The University of Queensland, Australia

Herbert Weissenboeck, University of Veterinary Medicine Vienna, Austria

Harshima Wijesinghe, University of Colombo, Sri Lanka

Andrew Williams, University of Copenhagen, Denmark

Scott Williams, Connecticut Agricultural Experiment Station, United States

Vyacheslav Yurchenko, University of Ostrava, Czech Republic

Xing Zhang, State Key Laboratory of Integrated Management of Pest Insects and Rodents, China

Ning Zhao, Chinese Center for Disease Control and Prevention, China

Bianca Zingales, University of Sao Paulo, Brazil

Annetta Zintl, University College Dublin, Ireland

Pascal Zumstein, University of Bern, Switzerland
